# Chronic Ataxic Neuropathy With Disialosyl Antibodies Responsive to Zanubrutinib

**DOI:** 10.1111/ene.70521

**Published:** 2026-02-24

**Authors:** Benedetta Tierro, Andrea Visentin, Alessandro Salvalaggio, Gabriella Cacchiò, Nicolò Danesin, Susanna Ruggero, Francesco Logullo, Chiara Briani

**Affiliations:** ^1^ Neurology Unit, Department of Neurosciences University of Padova Padova Italy; ^2^ Hematology Unit, Department of Medicine University of Padova Padova Italy; ^3^ Neurology Unit AST AP Mazzoni Hospital Ascoli Piceno Italy; ^4^ Neurology Unit Pesaro Hospital Pesaro Italy

**Keywords:** CANDA, CANOMAD, zanubrutinib

## Abstract

**Objectives:**

To describe a patient with chronic ataxic neuropathy with disialosyl antibodies (CANDA) with clinical and neurophysiological improvement after zanubrutinib therapy.

**Methods:**

A 62‐year‐old man started complaining of sensory symptoms in his hands and feet extending over time to the knees and elbows. Nerve conduction studies were consistent with diffuse sensory axonal ganglionopathy. Blood tests revealed an IgM 𝜆 paraprotein expression of Waldenström's macroglobulinemia. Anti‐MAG and anti‐neuronal antibodies were negative. Anti‐ganglioside IgM antibodies (GD1b; GD2, GD3, GT1a, GT1b, GQ1b) were positive. Neurological examination disclosed pinprick and tactile hypoesthesia with stocking‐glove distribution. The patient was responsive to intravenous immunoglobulin (IVIg) but reported end‐dose fluctuations. Rituximab was started but soon discontinued for clinical worsening, so IVIg therapy was resumed.

**Results:**

The patient was started on zanubrutinib, a second generation Bruton's tyrosine kinase (BTK) inhibitor, while continuing IVIg with global improvement and absence of end of dose efficacy. After 15 months, besides a clinical and hematological amelioration, a dramatic neurophysiological improvement also occurred. Antibodies to IgM GM1 and GM2 were not detectable, whereas anti‐disialosyl antibodies GD1a and GD1b persisted unchanged.

**Discussion:**

To the best of our knowledge, this is the first patient with Waldenström‐associated CANDA with clinical, hematological, and neurophysiological benefit after zanubrutinib therapy.

## 1 Introduction

CANOMAD (chronic ataxic neuropathy, ophthalmoplegia, IgM paraprotein, cold agglutinins, and disialosyl antibodies) or CANDA (chronic ataxic neuropathy with disialosyl antibodies) are immune‐mediated neuropathies associated with IgM monoclonal gammopathy and antibodies to disialosyl antigens [1].

They commonly respond to intravenous immunoglobulin (IVIg) or rituximab [2, 3], but due to the rarity of the diseases, controlled trials are lacking.

Association with hematological malignancies has been described, with Waldenström's macroglobulinemia (WM) being the most common [4].

We report on a WM‐associated CANDA patient who showed clinical, neurophysiological, and hematological improvement after therapy with the Bruton's tyrosine kinase (BTK) inhibitor zanubrutinib.

## 2 Case Report

In January 2024, a 62‐year‐old man came to our attention because of sensory symptoms in his hands and feet that started in 2018 and extended over time to knees and elbows. Nerve conduction studies detected a sensory axonal ganglionopathy at four limbs (Table 1).

**TABLE 1:** Blood exams and nerve conduction studies during IVIg (March 2022) and after zanubrutinib (July 2025).

	Baseline	+6 months	+15 months
Laboratory data			
WBC (10^9^/L)	10.7	8.2	8.6
ALC (10^9^/L)	4.91	3.88	3.48
Hb (g/L)	150	149	157
PLT (10^9^/L)	324	232	236
M‐protein (g/L)	2.7	2.5	2.3
FLC kappa (g/L)	1.85	4.07	2.61
FLC lambda (g/L)	1.73	2.75	1.81
IgG (mg/dL)	1473	1294	1247
IgA (mg/dL)	314	253	243
IgM (mg/dL)	294	170	150
Anti‐disialosyl antibodies titer (IgM)			
GM1	1/12800	1/25600	< 1/1600
GM2	1/1600	< 1/1600	< 1/1600
GD1a	1/3200	1/3200	1/3200
GD1b	1/100000	1/100000	1/100000

Electrophysiology	Date	DML ms	CMAP mV	MCV m/s	SNAP μV	SCV m/s	*F*‐wave ms
R median	2022	5.6	9.8	—	Absent	—	—
	2025	4.7	17	—	7 (dispersed)	43	—
L median	2022	4.9	d: 16.1 p: 13.7	—	—	—	29.3 (APB)
	2025	3.8	d: 18 p: 16.2	48	7 (dispersed)	37	31 (APB)
R ulnar	2022	3.5	14.4	—	Absent	—	—
	2025	3.2	13	—	Absent	—	—
L ulnar	2022	2.7	15	—	—	—	—
	2025	—	—	—	Absent	—	—
R radial	2022	—	—	—	—	—	—
	2025	—	—	—	4.3 (dispersed)	47	—
L radial	2022	—	—	—	—	—	—
	2025	—	—	—	7.8 (dispersed)	44	—
R sural	2022	—	—	—	Absent	—	—
	2025	—	—	—	Absent	—	—
L sural	2022	—	—	—	Absent	—	—
	2025	—	—		Absent	—	—
R deep peroneal	2022	5.4	d: 8.4 p: 7.1	45	—	—	43 (EDB)
	2025	4.7	d: 10.7 p: 8	46	—	—	52.4 (EDB)
L deep peroneal	2022	5.1	d: 12.2 p: 11.7	44	—	—	44.6 (EDB)
	2025	4.9	d: 11 p: 10	48	—	—	52.2 (EDB)
R superficial peroneal	2022	—	—	—	Absent	—	—
	2025	—	—	—	Absent	—	—
L superficial peroneal	2022	—	—	—	Absent	—	—
	2025	—	—	—	Absent	—	—

Abbreviations: ALC: absolute lymphocyte count; APB: abductor pollicis brevi; CMAP: compound muscle action potential; d: distal; DML: distal motor latency; EDB: extensor digitorum brevis; FLC: free light chains; Hb: hemoglobin; L: left; M‐protein: monoclonal protein; MCV: motor conduction velocity; p: proximal; PLT: platelets; R: right; SCV: sensory conduction velocity; SNAP: sensory nerve action potential; WBC: white blood cells.

Blood tests revealed an IgM lambda paraprotein expression of a WM from a bone marrow biopsy. Anti‐myelin‐associated glycoprotein (MAG) was negative.

Anti‐ganglioside antibodies revealed high titers of serum anti‐GD1a, anti‐GD1b, GD2, GD3, GT1a, GT1b, and GQ1b IgM. Cerebrospinal fluid exam was unremarkable.

The patient underwent IVIg therapy in January and February 2019.

Although IVIg responsive, the patient reported end of dose fluctuations. Rituximab was started but soon discontinued for clinical worsening so IVIg was resumed in March 2019. Due to the COVID‐19 pandemic, the IVIg were administered every 8 weeks with clear end of dose worsening.

A neurophysiological control in May 2023 confirmed sensory neuronopathy and revealed also bilateral carpal tunnel syndrome (Table 1).

When we first saw the patient (January 2024) he was on IVIg (30 g/day for 3 consecutive days each month) with end‐of‐dose effects (difficulty buttoning and writing, balance unsteadiness).

Neurological examination disclosed pinprick and tactile hypoesthesia with stocking‐glove distribution, and lower limbs areflexia. Total neuropathy score clinical version (TNSc) [5] was 9 (sensory symptoms 3, pin‐prick 3, deep tendon reflexes 3). A second bone marrow biopsy (February 2024) found clonal B neoplastic cell infiltration, MYD88^L265P^ mutation whereas CXCR4 non‐sense and frameshift mutations were absent. Blood exams found a monoclonal protein IgM/lambda (Table 1) while cryoglobulin, complement C3 and C4, and rheumatoid factor were not detected.

High titers of IgM anti‐GD1b (1:100000), GD1a (1:3200), GM2 (1:1600), GM1 (1:12800) (normal values < 1:1600) antibodies were present, confirming the diagnosis of CANDA associated‐WM.

In April 2024, the patient started zanubrutinib while continuing IVIg.

After 6 months, he reported global improvement (TNSc 2, score given by reduced ankle reflexes) and absence of IVIg end of dose efficacy.

In July 2025 (+15 months) the patient showed persistence of improvement, IgM decrease (Table 1), and no zanubrutinib side effects. Neurophysiological evaluation dramatically improved (Table 1). Antibodies to IgM GM1 and GM2 were not detectable, whereas antibodies to GD1a and GD1b persisted unchanged (as to the latter, it is difficult to determine when antibody titers above 1/100,000 dilution decline, because of the saturation effect).

Zanubrutinib, a second generation BTK inhibitor, is approved for treatment of several B‐cell malignancies including naive or relapsed/refractory WM with higher response rates and low adverse events. Zanubrutinib is currently under investigation for anti‐MAG antibody neuropathy.

To the best of our knowledge, this is the first WM‐associated CANDA treated with zanubrutinib with clinical, hematological, and neurophysiological benefit.

## Author Contributions

Benedetta Tierro and Alessandro Salvalaggio had a major role in the acquisition, analysis, and interpretation of data; contribution in drafting the manuscript for intellectual content. Gabriella Cacchiò had a major role in the acquisition, analysis, and interpretation of clinical data. Francesco Logullo performed the neurophysiological analyses and had a major role in the analysis and interpretation of the data. Andrea Visentin and Nicolò Danesin are the hematologists in charge of the patients, who had a major role in the acquisition, analysis, and interpretation of clinical data. Susanna Ruggero is the PhD in charge of the laboratory (antibody testing) analyses and had a major role in the analysis and interpretation of the data. Chiara Briani had a major role in the design and conceptualization of the study; coordinated the study; interpreted the data; and drafted the manuscript for intellectual content.

## Conflicts of Interest

The authors had no conflicts of interest.

## Data Availability

The data that support the findings of this study are available from the corresponding author upon reasonable request.
